# Prebiotic Potential of *Agave angustifolia* Haw Fructans with Different Degrees of Polymerization

**DOI:** 10.3390/molecules190812660

**Published:** 2014-08-19

**Authors:** José Rodolfo Velázquez-Martínez, Rina M. González-Cervantes, Minerva Aurora Hernández-Gallegos, Roberto Campos Mendiola, Antonio R. Jiménez Aparicio, Martha L. Arenas Ocampo

**Affiliations:** 1 Centro de Desarrollo de Productos Bióticos, Instituto Politécnico Nacional, P.O. Box 24, Yautepec 62730, Morelos, Mexico; E-Mails: rcamposm@ipn.mx (R.C.M.); aaparici@ipn.mx (A.R.J.A.); 2 Departamento de Sistemas Biológicos, Universidad Autónoma Metropolitana-Xochimilco, Calzada del Hueso 1100, Col. Villa Quietud, Delegación Coyoacán 04960, D.F., Mexico; E-Mail: gcrm4280@correo.xoc.uam.mx; 3 Escuela Nacional de Ciencias Biológicas, Instituto Politécnico Nacional, Prolongación de Carpio y Plan de Ayala s/n, Col. Santo Tomas, Delegación Miguel Hidalgo 11340, D.F., Mexico; E-Mail: mineaurora@gmail.com

**Keywords:** Bifidobacterium, Lactobacillus, Agave angustifolia Haw, prebiotic, fructans

## Abstract

Inulin-type fructans are the most studied prebiotic compounds because of their broad range of health benefits. In particular, plants of the *Agave* genus are rich in fructans. Agave-derived fructans have a branched structure with both β-(2→1) and β-(2→6) linked fructosyl chains attached to the sucrose start unit with a degree of polymerization (DP) of up to 80 fructose units. The objective of this work was to assess the prebiotic potential of three *Agave angustifolia* Haw fructan fractions (AFF) with different degrees of polymerization. The three fructan fractions were extracted from the agave stem by lixiviation and then purified by ultrafiltration and ion exchange chromatography: AFF1, AFF2 and AFF3 with high (3–60 fructose units), medium (2–40) and low (2–22) DP, respectively. The fructan profile was determined with high-performance anion exchange chromatography with pulsed amperometric detection (HPAEC-PAD), which confirmed a branched fructan structure. Structural elucidation was performed by Fourier Transform Infra-Red Spectroscopy. The AFF spectrum shows characteristic fructan bands. The prebiotic effect of these fractions was assessed *in vitro* through fermentation by *Bifidobacterium* and *Lactobacillus* strains. Four growth patterns were observed. Some bacteria did not grow with any of the AFF, while other strains grew with only AFF3. Some bacteria grew according to the molecular weight of the AFF and some grew indistinctly with the three fructan fractions.

## 1. Introduction

The genus *Agave* is endemic to America, and about 75% of the species are found in Mexico, considered the center of origin and diversity of the genus. Since pre-Columbian times, products made from these plants have been part of Mexican history and culture [1]. Today, only a few species of this genus are used, mainly in the manufacture of alcoholic beverages such as tequila (*A. tequilana* Weber), mezcal (*A. angustifolia* Haw, *A. asperrima* Jacobi, *A. weberi* Cela, *A. potatorum* Zucc and *A. salmiana* Otto) and bacanora (*A. angustifolia* Haw). *A. angustifolia* Haw is second, after *A. tequilana* Weber [2] in national production. Agave plants have a crassulacean acid metabolism (CAM) and the main products of photosynthesis are fructans, which are synthesized and stored in the stems, whose main function is storage, but they are also osmoprotectants during drought [3].

After starch, fructans are the most abundant nonstructural polysaccharides in Nature, present in about 15% of flowering plants. They are mixtures of oligomers and polymers of fructose linked by β-(2→1) and/or β-(2→6) links and synthesized from one molecule of sucrose. Fructans are classified according to their fructosyl bonds. Inulin and levan fructans have linear chains of fructose linked with β-(2→1) and β-(2→6) bonds, respectively. Graminans are branched fructans which have chains with both types of bonds β-(2→1) and β-(2→6), while neoseries have two linear β-(2→1) or β-(2→6)-linked fructosyl chains, one attached to the fructosyl residue of sucrose and the other attached to the glucosyl residue generating levan and inulin neoseries [4,5,6,7]. The genus *Agave* has branched fructans (graminan), and graminan neoseries structures with two branches have been identified. One branch is attached to the fructosyl residue of sucrose and the other attached to glucosyl, which has been called “agavins”. Agavins have a complex mixture of fructans with different DPs [7,8].

Due to the nature of the β links, fructans cannot be digested by the human upper digestive tract. It has also been demonstrated that inulin can be fermented by certain bacterial species of the intestinal microbiota. Therefore, it has been recognized as a prebiotic [9,10]. Prebiotics are carbohydrates resistant to human gastric digestion and are defined as a selective fermented ingredient that allows specific changes in the composition and/or activity of the gastrointestinal microbiota that benefits host well-being and health. Prebiotics act on the increase of biomass, regulating fecal transit in the colon, absorption of calcium and other minerals, production of endocrine peptides, immunity and resistance to infection, and lipid homeostasis. When involved with probiotic microorganisms, they improve the activity and composition of intestinal microbiota. The genera *Lactobacillus* and *Bifidobacterium* are predominant intestinal microbiota and some strains are recognized as probiotics that benefit health. Prebiotics have also been shown to have the ability to reduce intestinal infections, irritable bowel disease, colon cancer, osteoporosis and obesity [11,12].

Several studies indicate that fructan DP has an impact on fructan effectiveness as a prebiotic. Most studies find that fructans with a low degree of polymerization offer better stimulation of probiotic bacteria. However, others report that the prebiotic effect of fructans is indistinct of DP since some bacterial consortia show growth stimulation depending on the size of the fructan chain [10,13,14]. There are few reports on the prebiotic effect of *Agave* fructans. Márquez-Aguirre and coworkers report some health benefits using *Agave tequilana* fructans with a low DP. They found that it prevented body weight gain and fat tissue accumulation and reduced total cholesterol in an *in vivo* experiment using a diet-induced obesity model in mice [15]. The goal of this study was to determine, *in vitro*, the prebiotic effect of fructans, with different degrees of polymerization, extracted from *Agave angustifolia* Haw on strain of *Bifidobacteria* and *Lactobacilli*.

## 2. Results and Discussion

### 2.1. Agave Fructan Extraction and Purification

Crude extract was obtained by lixiviation using water as solvent [16]. Extract properties were 15.1 °Brix, pH 4.9, conductivity 1403 μs and 0.2% protein. After 10 KDa cut-off ultrafiltration, they were 15 °Brix, pH 5.02 and conductivity 1365 μs. This extract was used for further ion-exchange chromatography purification, resulting in a purified *Agave angustifolia* Haw fructan extract (PAFE), which was colorless, and had 11.4 °Brix, pH 6.09 and conductivity 18.5 μs, indicating that charged molecules were removed and most of the recovered molecules were fructans. This was confirmed by HPLC (data not shown) (Table 1) [17].

**Table 1:** Purification and fractionation monitoring of the *Agave angustifolia* Haw fructans.

	Purification				Fractionation		
	Crude Extract	Deproteinized UF 10 KDa	Ion-exchange Chromatography		AFF1 > 3 KDa	AFF2 3 a 1 KDa	AFF3 < 1 KDa
Conductivity μs	1403	1365	18.5		22	22	21
pH	4.9	5.02	6.09		6.2	6.1	6.2
Dissolved solids °Brix	15.1	15	11.4		19	21	22
Protein %	~0.2	-	-		-	-	-
Vol L	17	16	19.5		5.13	3.23	2.12
Total solids g	2567	2400	2223		974.7	678.3	466.4
				%	46	32	22

### 2.2. Agave Fructans Fractionation

**Figure 1:**
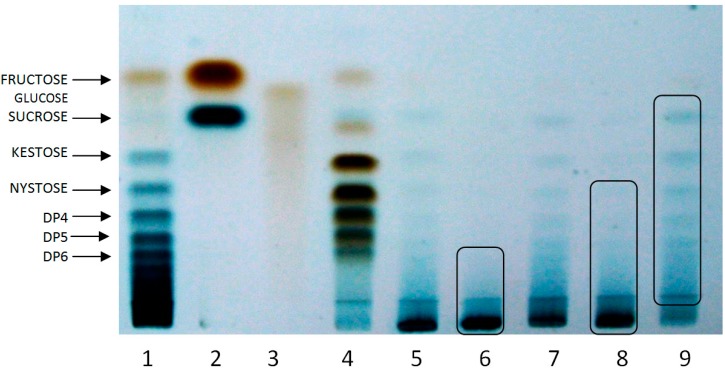
Thin Layer Chromatography (TLC) of Agave Fructose Fractions (AFF): 1 Chicory inulin, 2 Fructose + Sucrose, 3 Glucose, 4 FOS 2–7 DP, 5 PAFE, 6 retentate 3 KDa (AFF1), 7 Permeate 3 KDa, 8 retentate 1 KDa (AFF2) and 9 permeate 1 KDa (AFF 3).

Three fractions were obtained from PAFE: AFF1, AFF2 AND AFF3, considered fructan fractions with high, medium and low molecular weight, respectively, (Figure 1, lines 6, 8 and 9).

The pH of all fructan fractions was about 6.2 and conductivity around 22 μs. Total recovered fructans was 2119.4 g, considering that extraction from 17 kg material initial weight yielded about 12.5%, a value close to previously reported yield values for agave and chicory roots [18,19].

### 2.3. AFF Fructan Profile (DP)

**Figure 2:**
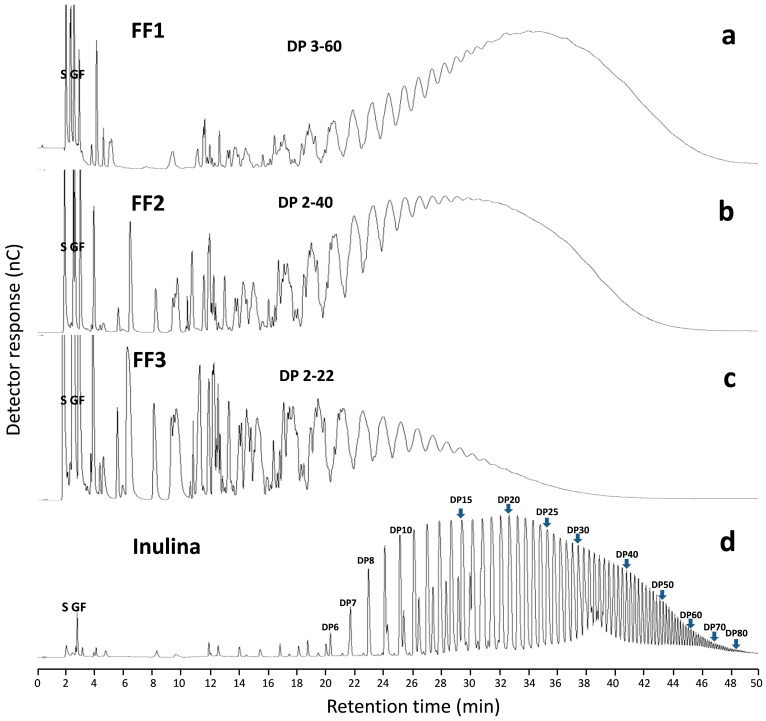
HPAEC-PAD chromatogram profiles. (**a**) AFF1, (**b**) AFF2, (**c**) AFF3, and (**d**) inulin profile. S, G and F refer to sucrose, glucose and fructose, respectively.

Fructan profiles of AFF from the HPAEC-PAD analysis (Figure 2a–c) are complex compared with chicory inulin profile (Figure 2d). This difference is mainly due to the nature of the linear structure of the fructan polymer contained in inulin-type fructans and the branched structure of the fructan chain in agave [20,21,22]. Mellado-Mojica and López [8] found that in *Agave tequilana* plants fructan molecular structures become more complex and DP was higher with plant age; they reported a large number of fructan isoforms: seven DP3 forms, eight DP4 and six DP5. In our study, samples for preparation of the fructan extract were taken from a 6-year-old agave stem, and our results were similar, with up to DP60 in AFF1 (Figure 2a). In another study [7], fructans were classified into three groups depending on DP and linkage-type abundance. *Agave angustifolia* fructans were categorized as branched neo-fructans and termed “agavin” to differentiate them from line-structured fructans called inulin. Moreover, observed DP results for each fraction were DP 3–60 for AFF1 (Figure 2a), DP 2–40 for AFF2 (Figure 2b) and DP 2–22 for AFF3 (Figure 2c). These results are in accordance with the TLC analysis mentioned above.

### 2.4. Molecular Recognition of Fructans Using Fourier Transform Infra-Red (FTIR) Spectroscopy

**Figure 3:**
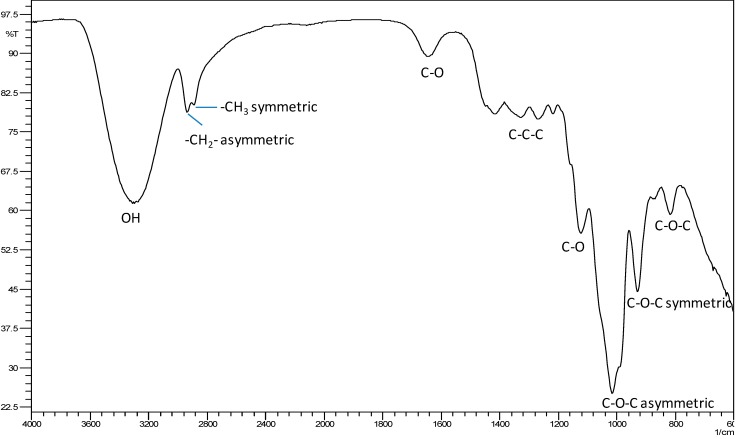
FTIR spectra of Agave fructan fraction (AFF1) from *A. angustifolia* Haw.

FTIR spectroscopy was used to evaluate the fructans’ structure. The FTIR spectrum of AFF1 is shown in Figure 3. Similar FTIR spectra were obtained for AFF2 and AFF3 (not shown). The broad absorbance band at 3600–3200 cm^−1^ is due to stretching of the hydroxyl groups, including carbohydrate and phenolic hydroxyl groups. The absorbance band at 3200–2800 cm^−1^ is attributed to C-H stretching and bending vibrations. In the spectral region, in the range of 1200–900 cm^−1^, we can observe a predominance of bands attributed to C-C, C-O stretching and C-O-H, C-O-C bending, characteristic of several oligo- and polysaccharides [23,24]. Each particular carbohydrate has a specific band in this region, which is within the so-called fingerprint region where the position and the intensity of the bands are specific for each sugar, so that identification is possible. On the other hand, the bands corresponding to protein in the 1550 cm^−1^ range are not observed, nor are nucleic acids observed in the 1250 cm^−1^ range or lipids in the 2030 cm^−1^ range. it is only carbohydrates that are observed in a pronounced band in the 1080 cm^−1^ range [25]. These results testify to the purity of the samples. A review of the literature shows that the FTIR spectrum of our results largely coincide with those obtained for artichoke, wheat bran, chicory and bamboo fructans [16,26,27,28].

### 2.5. Prebiotic Effect “In Vitro”

Some complex carbohydrates cannot be assimilated in the gastrointestinal tract and reach the colon. Here, if they promote the growth of beneficial bacteria, they are considered prebiotics. Prebiotics also stimulate health benefits in the host and fructans are known to be “good” promoters of probiotic bacteria mainly some strains of *Bifidobacterium* and *Lactobacillus*. Sources of fructans are therefore being tested to produce fructan products. We tested several species of *Bifidobacterium* and *Lactobacillus* for their capacity to metabolize fructans from agave. These bacteria had different capacities for fructan fraction assimilation. The ability to transport and metabolize different sizes of these molecules may depend on the nature of each strain [29,30]. Table 2 shows the different strains we used and cultivated in fermentation experiments with semi-synthetic medium. Each of the three AFF obtained by extraction-purification-fractionation was the sole carbon source; fructose was the fermentation control. Four groups of growth patterns that reveal AFF assimilation by probiotic strains were found.

**Figure 4:**
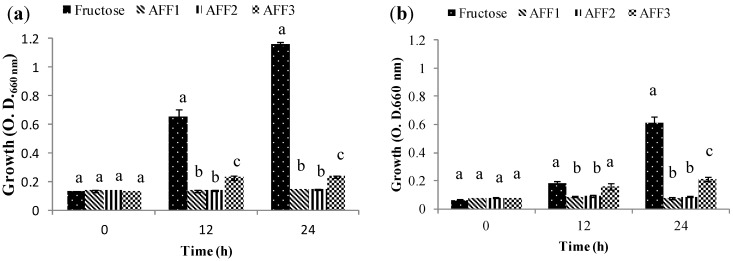
Growth (O.D.) of probiotic bacteria in Group 1 (G1) at 0, 12 and 24 h in semi-synthetic medium using AFF1, AFF2, AFF3 as the sole carbon source and fructose as fermentation control. (**a**) *Lactobacillus casei* subsp. rhamnosus ATCC 9595. (**b**) *Lactobacillus plantarum* 299 V.

Note: Means for the different carbon sources at the same times with the same letter are not significantly different (Duncan’s Multiple Range Test, *p* ≤ 0.05).

Group 1 includes those strains that are not able to use fructans as their sole carbon source regardless of the fructan DP. This was the case for *Lactobacillus casei* subsp. rhamnosus ATCC 9595 (Figure 4a) and *Lactobacillus plantarum* 299V (Figure 4b). *L. casei* could, however, grow well in the fructose control, while *L. plantarum* had moderate growth in same condition. These results are similar to those reported by Falony *et al.*, [31]; they describe a Group A whose strains are not able to grow on FOS-type inulin and suggest that these bacteria can survive in the gastrointestinal tract by using simple sugars generated by other bacteria that consume fructans.

**Table 2:** *Bifidobacterium* and *Lactobacillus* strains clustered in four groups (G), with different patterns of growth.

Strain	G1	G2	G3	G4
*B. adolecentis* ATCC 15703			X	
*B. animalis* ATCC 25527		X		
*B. bifidum* ATCC 29521				X
*B. breve* ATCC 15700		X		
*B. infantis* ATCC 17930			X	
*B. longum* ATCC 15707		X		
*B. lactis* DSM 10140		X		
*L.* casei subsp. rhamnosus ATCC 9595	X			
*L. paracasei* subsp. paracasei				X
*L. plantarum* 299V	X			

**Figure 5:**
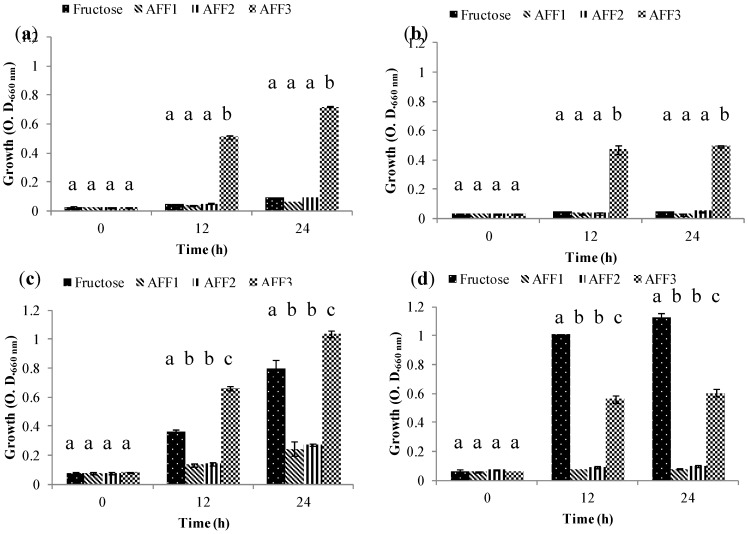
Growth (O.D.) of probiotic bacteria in Group 2 (G2) at 0, 12 and 24 h in semi-synthetic medium using AFF1, AFF2, AFF3 as the sole carbon source and fructose as fermentation control. (**a**) *Bifidobacterium lactis* DSM 10140. (**b**) *Bifidobacterium animalis* ATCC 25527. (**c**) *Bifidobacterium longum* ATCC 15707. (**d**) *Bifidobacterium breve* ATCC 15700.

NOTE: Means for the different carbon sources at the same times with the same letter are not significantly different (Duncan’s Multiple Range Test, *p* ≤ 0.05).

Group 2 includes those bacteria that grow only in the presence of low molecular weight fructans: (AFF3), *Bifidobacterium lactis* DSM10140 (Figure 5a), *Bifidobacterium animalis* ATCC 25527 (Figure 5b), *Bifidobacterium longum* ATCC 15707 (Figure 5c) and *Bifidobacterium* *breve* ATCC 15700 (Figure 5d). All four strains showed growth with AFF3 as their sole carbon source. For *B. animalis* and *B. longum*, growth is relatively low; this may be attributable to this particular fraction, which is a mix of diverse DP fructans and the quantity of DP 2–3 fructans may be reduced. Bifidobacteria have been shown to be specific for DP 2–3 fructans, and in the presence of DP 4 or higher its growth may have been affected [31,32]. Moreover, preference for FOS is characteristic of many *Bifidobacterium* species [33,34].

**Figure 6:**
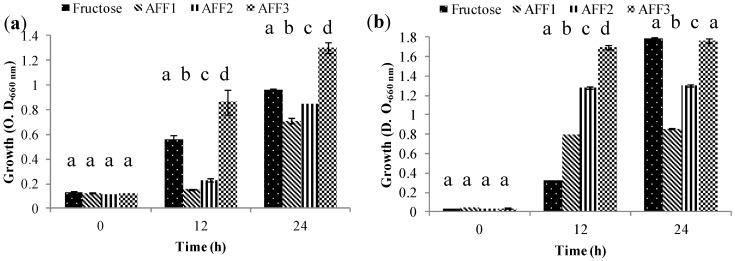
Growth (O.D.) of probiotic bacteria in Group 3 (G3) at 0, 12 and 24 h in semi-synthetic medium using AFF1, AFF2, AFF3 as the sole carbon source and fructose as fermentation control. (**a**) *Bifidobacterium adolecentis* ATCC 15703. (**b**) *Bifidobacterium infantis* ATCC 17930.

NOTE: Means for the different carbon sources at the same times with the same letter are not significantly different (Duncan’s Multiple Range Test, *p* ≤ 0.05).

Group 3 is represented by those strains that were able to grow using all fractions obtained as their carbon source but with a preference for low DP (AFF3), followed by medium DP (AFF2) and apparently a limited amount of high DP (AFF1) fructans (Figure 6). This group was represented by *Bifidobacterium adolescentis* ATCC 15703 (Figure 6a) and *Bifidobacterium infantis* ATCC 17930 (Figure 6b). This situation occurs when carbohydrate metabolism is more complex and cells assimilate preferably low DP fructans. When these are completely consumed, they start using medium DP fructans. Moderate growth in AFF1 was also observed; AFF1 contains DP3–60 and differed significantly from AFF3 and AFF2. This means that, although at first the fermentation strains used low DP fructans contained in this fraction, they finally used high DP fructans but less effectively. Similar results were found with *B. animalis* DN-173 010 [34].

**Figure 7:**
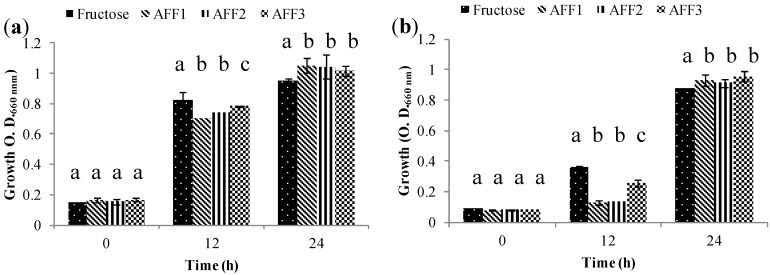
Growth (O.D.) of probiotic bacteria in the Group 4 (G4) at 0, 12 and 24 h in semi-synthetic medium using AFF1, AFF2, AFF3 as the sole carbon source and fructose as the fermentation control. (**a**) *Lactobacillus paracasei* subsp. paracasei. (**b**) *Bifidobacterium bifidum* ATCC 29521.

NOTE: Means for the different carbon sources at the same times with the same letter are not significantly different (Duncan’s Multiple Range Test, *p* ≤ 0.05).

Group 4 comprises strains that grow regardless of the fructan fraction carbon source. For probiotic bacteria such as *Lactobacillus paracasei* subsp. paracasei (Figure 7a) and *Bifidobacterium bifidum* ATCC 29521 (Figure 7b) growth reaches the same optical density, although at different times. This capacity of indiscriminate fructan consumption is attributed to the synthesis of intra and extracellular hydrolytic enzymes (β-fructofuranosidases) supported by specific transport systems for fructans of diverse molecular weights [13,35].

Falony *et al.*, [31] conducted an extensive study with 18 bifidobacterial strains and found four groups (A,B,C,D). Groups 1, 2 and 3 of our work share similar results with groups A, B and C, respectively. However, unlike group four in our study, none of the strains used by Falony and co-workers was able to use high DP fructans. This difference could reside in that we obtained fructans from agave extract, in contrast to those from the inulin used in the referred study. Another explanation could be simply that strains used in the two studies are different, and each strain has its particular ability for fructan consumption.

## 3. Experimental Section 

### 3.1. Chemical Reagents

Butanol, methanol, acetic acid, K_2_HPO_4_, MgCl_2_, ZnSO_4_, CaCl_2_, FeCl_3_, MgSO_4_, KH_2_PO_4_, NaHCO_3_, NaCl were acquired from J.T. Baker^®^ (Mex, México), Tween 80, anisaldehyde, cysteine, casein enzymatic hydrolysate were obtained from Sigma-Aldrich^®^ (St. Louis, MO, USA) and MRS medium was obtained from BD Difco^TM^ (Le Pont de Claix, France), yeast nitrogen base was obtained from BD Difco^TM^ (Sparks, MD, USA). Sodium thioglycolate was obtained from Dibico^®^ (Mex, México). Glucose, casein peptone, soy peptone and yeast extract were acquired from BD Bioxon^TM^ (Mex, México). CO_2_/N_2_ was purchased from INFRA (Mex, Mexico). The glucose, fructose, sucrose and nystose standards were obtained from Sigma-Aldrich^®^.

### 3.2. Extraction of Agave angustifolia Haw Fructans

#### 3.2.1. Agave Stems

The stem of a 6-year-old *A. angustifolia* Haw was collected in Barranca Honda in the Municipality Yautepec, state of Morelos, Mexico (18°81'99''N, 99°10'75''W). It was washed with water and reduced to a particle size of about one centimeter.

#### 3.2.2. Extraction

Samples were stirred continuously in water at 80 °C for 1 hour using an agave:water ratio of 1:1 (w/v). The aqueous extract was filtered through a 200 mesh and then through a one-micron filter. The crude extract was stored at 4 °C until use [18,36].

### 3.3. Purification of Agave angustifolia Haw fructans

The crude extract was ultra-filtered using a 10 KDa MWCO hollow fiber cartridge (Amicon^TM^, Merck Millipore, Darmstadt, Alemania) to remove proteins. The acid components, pigments, dyes, minerals and other ionic molecules were removed from the crude extract by ion-exchange chromatography, using a cationic (Amberlite™ IRI 20 Na) and anionic resin (Amberlite™ FPA90 Cl); the effluent without ionic molecules was monitored by index of refraction and collected as purified extract [15,37,38]. Total fructans were determined by anion-exchange high-performance liquid chromatography (HPLC). HPLC conditions included an Aminex HPX- 87C column (Bio-Rad, Hercules, CA, USA), deionized water at 85 °C as the mobile phase, flow 0.5 mL/min and a refractive index detector [39].

### 3.4. Agave Fructans Fractionation

The purified extract was fractionated by ultrafiltration with a tangential flow filtration system (Cogent M1^®^, Merck Millipore, Darmstadt, Alemania) with 1 and 3 KDa MWCO cassettes (0.2 m^2^) at transmembrane pressure of 40 psi. The purified extract was ultrafiltered with 3 KDa MWCO cassette. Retentate (AFF1 > 3 KDa) was stored at 4 °C and permeate of 3 KDa MWCO was ultrafiltered with 1 KDa MWCO cassette. Retentate (AFF2 1 to 3 KDa) and permeate (AFF3 < 1 KDa) of 1 KDa MWCO were stored at 4 °C. Three fractions (AFF1, AFF2 and AFF3) were lyophilized and stored in amber bottles for subsequent use [15,40]. Purification and fractionation processes were monitored through the following determinations: Protein (Bradford method), pH, conductivity (µs) and soluble solids (°Brix).

#### Thin Layer Chromatography (TLC)

In order to follow the purification and fractionation of agave fructans, TLC was used. A total of 20 µL of an 80 mg/mL fructan solution (40 µg) was applied per line with a Linomat 5 applier (CAMAG^TM^, Muttenz, Switzerland) on HPTLC plates (NH_2_ F254). Plates were developed three times with butanol:methanol:water:acetic acid (50:25:20:1) and dried at room temperature. Samples were fixed at 180 °C for 10 min. Plates were sprayed with ethanol-anisaldehyde-sulfuric acid (18:1:1) and visualized at 120 °C.

### 3.5. AFF Fructan Profile (DP)

AFF profiles were constructed by HPAEC-PAD using a Dionex^TM^ ISC-5000^TM^ system (Thermo Scientific^TM^, Waltham, MA, USA) with a single pump (SP) and an electro chemical detector (ED). Twenty µL of sample at a concentration of 5 mg/mL was injected into the CarboPac PA200 column (4 × 50 mm) in series with a CarboPac PA200 guard column (3 × 50 mm) [41]. Samples were separated with the gradient elution program described in Table 3 [42,43]. Glucose, fructose, sucrose and nystose were identified by comparing peak retention times with their commercial standards.

**Table 3:** HPAEC-PAD gradient program for separating fructans and simple sugars ^a^.

Time (min)	Flow (mL/min)	Step
0–5	0.42	100% A
5–44	0.42	Gradient to 50% A/50% B
44–50	0.42	Isocratic at 50% A/50% B
50–53	0.35	Gradient 50% C/50% D
53–60	0.35	Isocratic at 50% C/50% D
60–70	0.35	Gradient to 100% A
70–80	0.35	100% A
80–90	0.42	100% A

ª Column oven temperature = 28 °C. Solvent A = 0.1 M NaOH; solvent B = 1 M NaOAc in 0.1 M NaOH; solvent C = water; solvent D = 200 mM NaOH. All gradients were linear.

### 3.6. Structure of Fructan from A. angustifolia Haw

The structure of *Agave angustifolia* Haw fructans was evaluated in the three fractions (AFF1, AFF2, AFF3) by Fourier Transform Infra-Red (FTIR) spectroscopy on an IRAffinity-1 FTIR spectrophotometer (Shimadzu, Kyoto, Japan) with Attenuated Total Reflection sampling accessories MIRacle ATR (PIKE Technologies, Madison, WI) with a ZnSe crystal. FTIR spectra were obtained on the spectral region range from 4000–600 cm^−1^ with resolution 2 cm^−1^ and 128 scans [23,27].

### 3.7. Prebiotic Effect “In Vitro”

#### 3.7.1. Media

Strains were activated in MRS medium supplemented with 0.5 g/L cysteine (MRS-cys). Inoculum was standardized with TPY-fru-cys, medium (TPY-cys) prepared with 5 g/L glucose, 10 g/L casein peptone, 5 g/L soy peptone, 2.5 g/L yeast extract, 0.5 g/L L-cysteine, 2 g/L K_2_HPO_4_, 0.5 g/L MgCl_2_, 0.25 g/L ZnSO_4_, 0.15 g/L CaCl_2_, 0.03 g/L FeCL_3_, 1 mL/L Tween 80 and adjusted to pH 7.0. To prepare TPY-fru-cys 5 g/L glucose was substituted by 5 g/L fructose. Fermentation was carried out with a semisynthetic medium with a minimal carbon source for supplementation with the different carbon sources to be monitored. Semisynthetic medium was prepared using 6.7 g/L yeast nitrogen base, 0.5 g/L l-cysteine, 1mL/L Tween 80 and 40% [v/v] salt solution consisting of 0.2 g/L CaCl_2_, 0.2 g/L MgSO_4_, 1 g/L K_2_HPO_4_, 1 g/L KH_2_PO_4_, 10 g/L NaHCO_3_, 2 g/L NaCl, 0.5 g/L casein enzymatic hydrolysate, 0.05 g/L sodium thioglycolate [44]. Monitored carbon sources were fructose, AFF1, AFF2 and AFF3.

#### 3.7.2. Microorganisms 

*Bifidobacterium adolecentis* ATCC 15703, *Bifidobacterium animalis* ATCC 25527, *Bifidobacterium bifidum* ATCC 29521, *Bifidobacterium breve* ATCC 15700, *Bifidobacterium infantis* ATCC 17930, *Bifidobacterium longum* ATCC 15707, *Bifidobacterium lactis* DSM 10140, *Lactobacillus casei* subsp. *rhamnosus* ATCC 9595 and *Lactobacillus paracasei* subsp. paracasei were obtained from the stock culture collection of Biotechnology Laboratory (Universidad Autonóma Metropolitana-Xochimilco, UAM-Xoc, México). Lactobacillus plantarum 299V was obtained from a lyophilized culture (Protansitus LP produced by Salvat in Barcelona, Spain). Strains were stored in 20% (v/v) glycerol at −80 °C prior to fermentation. Gram staining was performed in order to ensure all cultures were pure and not contaminated during storage. All bacterial strains before fermentation were activated by culturing in MRS-cys for 12 h and inoculum was standardized with two fermentations in TPY-fru-cys medium for 10 to 12 h, except for *Lactobacillus paracasei* subsp. *paracasei*, which was standardized with TPY-cys medium. Fermentations were carried out under anaerobic conditions at 37 °C and shaken at 200 rpm. All media were flushed with CO_2_/N_2_. For the anaerobic condition, flasks were capped with stoppers, sealed with aluminum rings and later sterilized at 121 °C.

#### 3.7.3. Fermentation from Fructans of *Agave angustifolia* Haw

Fermentation was conducted in semisynthetic medium using fructose, FFA1, FFA2 and FFA3 as carbon sources (5 g/L). Carbon sources were sterilized by filtration and injected into the flasks under sterile conditions. Fermentations were carried out under anaerobic condition at 37 °C and shaken at 200 rpm. Optical density was tested every 0, 12 and 24 h of incubation period. This reflected the growth of bacteria in different carbon sources. Duplicate samples were measured spectrophotometrically using a UV-visible spectrophotometer at 660 nm.

### 3.8. Statistical Analysis

Data obtained were analyzed using one-way ANOVA. Duncan’s Multiple range Test was applied to determine significant differences between carbon sources at *p* ≤ 0.05.

## 4. Conclusions

Extracts from six-year-old *Agave angustifolia* Haw have high molecular weight and branched fructans. With the process of extraction-purification-fractionation designed in this work, 3 fructan fractions were separated: AFF1, PD3–60; AFF2, PD2–40 and AFF3 PD2–22. These fractions used as carbon sources stimulated growth of probiotic bacteria in a different manner depending on the strain used in fermentation experiments. Of the four fermentative groups identified, AFF3 was the preferred carbon source for probiotic bacteria, showing high potential use as a prebiotic. Even though AFF1 and AFF2 are not readily assimilated as carbohydrate sources by most bacteria used in this study, we found some strains that could assimilate these fructan fractions, opening an interesting research path in the search for new efficient hydrolytic enzymes for prebiotic production.

## References

[1] García A. (2007). Los agaves de México. Ciencias.

[2] Trueba L.A.C. (2007). Los destilados de agave en méxico y su denominación de origen. Ciencias.

[3] Wang N., Nobel P.S. (1998). Phloem transport of fructans in the crassulacean acid metabolism species agave deserti. Plant Physiol..

[4] Vijn I., Smeekens S. (1999). Fructan: More than a reserve carbohydrate?. Plant Physiol..

[5] Ritsema T., Smeekens S. (2003). Fructans: Beneficial for plants and humans. Curr. Opin. Plant Biol..

[6] Waleckx E., Gschaedler A., Colonna-Ceccaldi B., Monsan P. (2008). Hydrolysis of fructans from *Agave tequilana* weber var. Azul during the cooking step in a traditional tequila elaboration process. Food Chem..

[7] Mancilla-Margalli N.A., López M.G. (2006). Water-soluble carbohydrates and fructan structure patterns from agave and dasylirion species. J. Agric. Food Chem..

[8] Mellado-Mojica E., López M.G. (2012). Fructan metabolism in a. Tequilana weber blue variety along its developmental cycle in the field. J. Agric. Food Chem..

[9] Kolida S., Gibson G.R. (2007). Prebiotic capacity of inulin-type fructans. J. Nutr..

[10] Biedrzycka E., Bielecka M. (2004). Prebiotic effectiveness of fructans of different degrees of polymerization. Trends Food Sci. Tech..

[11] Roberfroid M. (2007). Prebiotics: The concept revisited. J. Nutr..

[12] Saulnier D.M.A., Spinler J.K., Gibson G.R., Versalovic J. (2009). Mechanisms of probiosis and prebiosis: Considerations for enhanced functional foods. Curr. Opin. Biotech..

[13] Van de Wiele T., Boon N., Possemiers S., Jacobs H., Verstraete W. (2007). Inulin-type fructans of longer degree of polymerization exert more pronounced *in vitro* prebiotic effects. J. Appl. Microbiol..

[14] Pompei A., Cordisco L., Raimondi S., Amaretti A., Pagnoni U.M., Matteuzzi D., Rossi M. (2008). *In vitro* comparison of the prebiotic effects of two inulin-type fructans. Anaerobe.

[15] Márquez-Aguirre A.L., Camacho-Ruiz R.M., Arriaga-Alba M., Padilla-Camberos E., Kirchmayr M.R., Blasco J.L., González-Avila M. (2013). Effects of agave tequilana fructans with different degree of polymerization profiles on the body weight, blood lipids and count of fecal lactobacilli/bifidobacteria in obese mice. Food Function.

[16] López-Molina D., Navarro-Martínez M.D., Rojas-Melgarejo F., Hiner A.N.P., Chazarra S., Rodríguez-López J.N. (2005). Molecular properties and prebiotic effect of inulin obtained from artichoke (*Cynara scolymus* L.). Phytochemistry.

[17] Yang Z., Hu J., Zhao M. (2011). Isolation and quantitative determination of inulin-type oligosaccharides in roots of morinda officinalis. Carbohydr. Polym..

[18] Ávila-Fernández Á, Galicia-Lagunas N., Rodríguez-Alegría M.E., Olvera C., López-Munguía A. (2011). Production of functional oligosaccharides through limited acid hydrolysis of agave fructans. Food Chem..

[19] López M.G., Urıas-Silvas J., Shiami N., Benkeblia N., Ondera S. (2007). Agave Fructans as Prebiotics. Advances in Fructooligosaccharides Research.

[20] Jenkins C.L.D., Lewis D., Bushell R., Belobrajdic D.P., Bird A.R. (2011). Chain length of cereal fructans isolated from wheat stem and barley grain modulates *in vitro* fermentation. J. Cereal Sci..

[21] Pavis N., Chatterton N., Harrison P., Baumgartner S., Praznik W., Boucaud J., Prud’Homme M. (2001). Structure of fructans in roots and leaf tissues of lolium perenne. New Phytol..

[22] Ravenscroft N., Cescutti P., Hearshaw M.A., Ramsout R., Rizzo R., Timme E.M. (2009). Structural analysis of fructans from agave americana grown in south africa for spirit production. J. Agric. Food Chem..

[23] Grube M., Bekers M., Upite D., Kaminska E. (2002). Infrared spectra of some fructans. J. Spectroscopy.

[24] Fariña J., Viñarta S., Cattaneo M., Figueroa L. (2009). Structural stability of sclerotium rolfsii atcc 201126 β-glucan with fermentation time: A chemical, infrared spectroscopic and enzymatic approach. J. Appl. Microbiol..

[25] Nikonenko N., Buslov D., Sushko N., Zhbankov R. (2005). Spectroscopic manifestation of stretching vibrations of glycosidic linkage in polysaccharides. J. Mol. Struct..

[26] Wang J., Yuan X., Sun B., Cao Y., Tian Y., Wang C. (2009). On-line separation and structural characterisation of feruloylated oligosaccharides from wheat bran using hplc-esi-msn. Food Chem..

[27] Azmi A.F.M.N., Mustafa S., Hashim D.M., Manap Y.A. (2012). Prebiotic activity of polysaccharides extracted from gigantochloa levis (buluh beting) shoots. Molecules.

[28] Wu X.Y., Lee P.I. (2000). Preparation and characterization of inulin ester microspheres as drug carriers. J. Appl. Polym. Sci..

[29] Bolado M., Acedo F. (2006). Sugar catabolism in bifidobacteria. Salus.

[30] Kaplan H., Hutkins R.W. (2003). Metabolism of fructooligosaccharides by lactobacillus paracasei 1195. Appl. Environ. Microbiol..

[31] Falony G., Lazidou K., Verschaeren A., Weckx S., Maes D., De Vuyst L. (2009). *In vitro* kinetic analysis of fermentation of prebiotic inulin-type fructans by bifidobacterium species reveals four different phenotypes. Appl. Environ. Microbiol..

[32] Van der Meulen R., Makras L., Verbrugghe K., Adriany T., De Vuyst L. (2006). *In vitro* kinetic analysis of oligofructose consumption by bacteroides and bifidobacterium spp. indicates different degradation mechanisms. Appl. Environ. Microbiol..

[33] Amaretti A., Bernardi T., Tamburini E., Zanoni S., Lomma M., Matteuzzi D., Rossi M. (2007). Kinetics and metabolism of bifidobacterium adolescentis mb 239 growing on glucose, galactose, lactose, and galactooligosaccharides. Appl. Environ. Microbiol..

[34] Van der Meulen R., Avonts L., De Vuyst L. (2004). Short fractions of oligofructose are preferentially metabolized by bifidobacterium animalis dn-173 010. Appl. Environ. Microbiol..

[35] Makras L., van Acker G., De Vuyst L. (2005). Lactobacillus paracasei subsp. Paracasei 8700:2 degrades inulin-type fructans exhibiting different degrees of polymerization. Appl. Environ. Microbiol..

[36] Lingyun W., Jianhua W., Xiaodong Z., Da T., Yalin Y., Chenggang C., Tianhua F., Fan Z. (2007). Studies on the extracting technical conditions of inulin from jerusalem artichoke tubers. J. Food Engin..

[37] Hincha D.K., Livingston Iii D.P., Premakumar R., Zuther E., Obel N., Cacela C., Heyer A.G. (2007). Fructans from oat and rye: Composition and effects on membrane stability during drying. Biochimica et Biophysica Acta (BBA)–Biomembranes.

[38] Yamazaki H., Matsumoto K. (1994). Purification of jerusalem artichoke fructans and their utilisation by bifidobacteria. J. Sci. Food Agric..

[39] Michel-Cuello C., Ortiz-Cerda I., Moreno-Vilet L., Grajales-Lagunes A., Moscosa-Santillan M., Bonnin J., González-Chávez M.M, Ruiz-Cabrera M. (2012). Study of enzymatic hydrolysis of fructans from agave salmiana characterization and kinetic assessment. Sci. World J..

[40] Chandrashekar P.M., Prashanth K.V.H., Venkatesh Y.P. (2011). Isolation, structural elucidation and immunomodulatory activity of fructans from aged garlic extract. Phytochemistry.

[41] Corradini C., Cavazza A., Bignardi C. (2012). High-performance anion-exchange chromatography coupled with pulsed electrochemical detection as a powerful tool to evaluate carbohydrates of food interest: Principles and applications. Inter. J. Carbohydr. Chem..

[42] Kagan I.A., Kirch B.H., Thatcher C.D., Strickland J.R., Teutsch C.D., Elvinger F., Pleasant R.S. (2011). Seasonal and diurnal variation in simple sugar and fructan composition of orchardgrass pasture and hay in the piedmont region of the united states. J. Equine Vet. Sci..

[43] Willems J.L., Low N.H. (2012). Major carbohydrate, polyol, and oligosaccharide profiles of agave syrup. Application of this data to authenticity analysis. J. Agric. Food Chem..

[44] González R., Klaassens E.S., Malinen E., De Vos W.M., Vaughan E.E. (2008). Differential transcriptional response of bifidobacterium longum to human milk, formula milk, and galactooligosaccharide. Appl. Environ. Microbiol..

